# Valedictory

**Published:** 1888-12

**Authors:** 


### Editorial.

---

### VALEDICTORY.

With this number closes Volume IX of the INDEPENDENT PRACTITIONER, and with it end our first six months' work in dental journalism. Hardly more than an introduction. If we have succeeded in satisfying our readers thus far we shall be much pleased, for our summer's work has been a very trying one. Our editorials have been written on trains and in hotels without books of reference. We have been haunted with proof in our journeys in many cities and states, which has been corrected under very great disadvantages.

We are fully aware that some typographical errors have been overlooked, a few grammatical slips have crept in, and in some instances some loose writing has been allowed to pass unnoticed. Our only apology was our lack of time to carefully consider every detail. The last six numbers of the journal have been the result of our unaided efforts, and whatever of error or shortcomings is therein contained is ours and we must accept your criticism. We have not, unfortunately, a trained corps of assistants upon whom we can depend to carry on matters while we are away, as have some of the other journals, but must depend upon ourselves. Had we known that so much of the work would have fallen upon our shoulders unaided, we should never have made arrangements to "swing the circuit" of the dental meetings for the summer, let alone to take part in their programmes. In addition to the other drawbacks, the change of publishers has been added. We are now, however, fully established in our new quarters, and promise much better work for Volume X than has been done on Volume IX. The business of the Journal is in a most prosperous condition. Many new subscribers have been added the past summer, and the outlook for the future is very bright. What was termed "editorial boasting" in our first announcement has become a fruition, and the first of the year will see the INTERNATIONAL DENTAL JOURNAL successfully launched upon the sea of journalism.

On account of the change of name we have thought best to invite Dr. Barrett, former editor, and who has now accepted the position of associate editor to add a few words of retrospect in regard to the INDEPENDENT PRACTITIONER, and will therefore refrain from further notice until the beginning of the new volume, when the purposes of the journal will be more fully set forth.

